# The GRPR Antagonist [^99m^Tc]Tc-maSSS-PEG_2_-RM26 towards Phase I Clinical Trial: Kit Preparation, Characterization and Toxicity

**DOI:** 10.3390/diagnostics13091611

**Published:** 2023-05-02

**Authors:** Ayman Abouzayed, Jesper Borin, Fanny Lundmark, Anastasiya Rybina, Sophia Hober, Roman Zelchan, Vladimir Tolmachev, Vladimir Chernov, Anna Orlova

**Affiliations:** 1 Department of Medicinal Chemistry, Uppsala University, 751 83 Uppsala, Sweden; ayman.abouzayed@ilk.uu.se (A.A.); fanny.lundmark@ilk.uu.se (F.L.); 2 Department of Protein Science, KTH Royal Institute of Technology, 114 17 Stockholm, Sweden; jborin@kth.se (J.B.); sophia@kth.se (S.H.); 3 Department of Nuclear Medicine, Cancer Research Institute, Tomsk National Research Medical Center, Russian Academy of Sciences, 634009 Tomsk, Russia; rybina@onco.tnimc.ru (A.R.); zelchan@onco.tnimc.ru (R.Z.); chernov@tnimc.ru (V.C.); 4 Research Centrum for Oncotheranostics, Research School of Chemistry and Applied Biomedical Sciences, Tomsk Polytechnic University, 634050 Tomsk, Russia; 5 Department of Immunology, Genetics and Pathology, Uppsala University, 752 37 Uppsala, Sweden; vladimir.tolmachev@igp.uu.se; 6 Science for Life Laboratory, Uppsala University, 752 37 Uppsala, Sweden

**Keywords:** prostate cancer, GRPR antagonist, technetium-99m, sterility, toxicity testing

## Abstract

Gastrin-releasing peptide receptors (GRPRs) are overexpressed in the majority of primary prostate tumors and in prostatic lymph node and bone metastases. Several GRPR antagonists were developed for SPECT and PET imaging of prostate cancer. We previously reported a preclinical evaluation of the GRPR antagonist [^99m^Tc]Tc-maSSS-PEG_2_-RM26 (based on [D-Phe^6^, Sta^13^, Leu^14^-NH_2_]BBN(6-14)) which bound to GRPR with high affinity and had a favorable biodistribution profile in tumor-bearing animal models. In this study, we aimed to prepare and test kits for prospective use in an early-phase clinical study. The kits were prepared to allow for a one-pot single-step radiolabeling with technetium-99m pertechnetate. The kit vials were tested for sterility and labeling efficacy. The radiolabeled by using the kit GRPR antagonist was evaluated in vitro for binding specificity to GRPR on PC-3 cells (GRPR-positive). In vivo, the toxicity of the kit constituents was evaluated in rats. The labeling efficacy of the kits stored at 4 °C was monitored for 18 months. The biological properties of [^99m^Tc]Tc-maSSS-PEG_2_-RM26, which were obtained after this period, were examined both in vitro and in vivo. The one-pot (gluconic acid, ethylenediaminetetraacetic acid, stannous chloride, and maSSS-PEG_2_-RM26) single-step radiolabeling with technetium-99m was successful with high radiochemical yields (>97%) and high molar activities (16–24 MBq/nmol). The radiolabeled peptide maintained its binding properties to GRPR. The kit constituents were sterile and non-toxic when tested in living subjects. In conclusion, the prepared kit is considered safe in animal models and can be further evaluated for use in clinics.

## 1. Introduction

Prostate cancer is one of the most commonly diagnosed cancers and is the cause of many cancer-related deaths [1]. Among the explored prostate cancer cell markers are the prostate-specific membrane antigen (PSMA) and the gastrin-releasing peptide receptor (GRPR) [2], which are also expressed in other malignancies, including breast cancer [3,4].

Numerous PSMA-targeting radiotracers have been developed for positron emission tomography (PET) and single-photon emission computed tomography (SPECT), resulting in several approved radiotracers both for diagnosis and therapy of PSMA-expressing prostate cancer [5]. These radiotracers rely on the high expression of PSMA in the majority of prostate cancer cells, especially in the late stages of the disease, providing an improved diagnosis and monitoring of therapeutic response in comparison with the other available diagnostic and therapeutic options. There are several limitations of targeting PSMA, which include PSMA-negative prostate tumors [6]. Thus, it is necessary to develop and use radiotracers that target other markers, which include GRPR.

GRPR expression is found in 63–100% of primary prostate tumors and in the majority of lymph node and bone metastases [7,8,9]. Additionally, its expression in the prostate is mainly limited to malignant cells and has been found to be higher in the earlier stages of prostate cancer [10]. Therefore, we have selected and evaluated GRPR as a potential target for diagnostics and therapeutics of prostate cancer.

Radiolabeled GRPR antagonists have been proposed to have advantages over GRPR agonists in that they evade GRPR activation, which can stimulate cell proliferation and growth [11] and lead to GRPR downregulation [12], among other unwanted effects. In addition to this, radiolabeled GRPR antagonists were found to perform similarly to GRPR agonists, in imaging GRPR expression in vivo, if not better [13]. Various GRPR antagonists have been developed and evaluated in preclinical and early-phase clinical studies, showing great promise for imaging of GRPR-expressing prostate cancer [14].

Technetium-99m is one of the most commonly used radionuclides in nuclear medicine, which is attributed to its optimal energy of emitted photons, sufficiently long half-life, and to its feasibility of being acquired by elution from a ^99^Mo/^99m^Tc generator, enabling the acquisition of technetium-99m at a low cost. SPECT cameras, with their advantages and pitfalls, are still more widely available than PET cameras [15,16]. Therefore, radiotracers suitable for SPECT imaging will have a wider outreach and thereby benefit more patients in the near future; hence, several GRPR antagonists labelled with technetium-99m were developed [17,18,19].

For the aforementioned reasons, our group recently reported the development and preclinical evaluation of the GRPR antagonist maSSS-PEG_2_-RM26 [20], consisting of a mercaptoacetyl-triserine amino acid sequence linked to the GRPR antagonist RM26 [D-Phe^6^, Sta^13^, Leu^14^-NH_2_]BBN(6–14) [21] via a diethylene glycol linker. We used an amino acid-based N_3_S chelator formed by mercaptoacetyl-triserine for the labelling of peptide with freshly eluted pertechnetate (^99m^TcO_4_^−^) [22,23] that enabled single-step labeling. [^99m^Tc]Tc-maSSS-PEG_2_-RM26 demonstrated strong affinity for GRPR along with favorable biodistribution and dosimetry, showing promise for SPECT imaging of GRPR-positive prostate cancer.

Following our recent report on the development of [^99m^Tc]Tc-maSSS-PEG_2_-RM26, we aimed herein to prepare kits for single-step technetium-99m labeling of maSSS-PEG_2_-RM26 to be used in an early-phase clinical study. We also aimed to test the radiolabeling and target binding of [^99m^Tc]Tc-maSSS-PEG_2_-RM26 after kit preparation in addition to testing the sterility and toxicity of the kit constituents. 

Furthermore, we wanted to study the effect of long-term (18 months) storage of the kit formulation on the radiolabeling of maSSS-PEG_2_-RM26 and evaluate its in vitro and in vivo binding to GRPR after storage. 

## 2. Materials and Methods

Gluconic acid sodium salt and ethylenediaminetetraacetic acid (EDTA) were purchased from Sigma-Aldrich (St. Louis, MO, USA), stannous chloride was purchased from Fluka Chemika (Buchs, Switzerland), and PC-3 cells were purchased from ATCC (Rockville, MD, USA). Rosewell Park Memorial Institute (RPMI) 1640 medium supplemented with L-Glutamine was purchased from Biowest (Nuaillé, France), fetal bovine serum was purchased from Sigma-Aldrich (St. Louis, MO, USA), and penicillin-streptomycin (10,000 U/mL penicillin and 10,000 μg/mL streptomycin) and trypsin-EDTA were purchased from Biochrom AG (Berlin, Germany). 

The glass vials used for kit preparation were adaptiQ^®^ vials, purchased from Schott AG (Mainz, Germany), the stoppers used were 20 mm Serum NovaPure^®^ stoppers and the seals were 20 mm Flip-Off^®^ CCS seals, both purchased from West Pharmaceutical Services (West Whiteland Township, PA, USA). Technetium-99m pertechnetate (^99m^TcO_4_^−^) was eluted from an Ultra-TechneKow^®^ FM ^99^Mo/^99m^Tc generator (Curium, Petten, The Netherlands). Radioactivity measurements for cell experiments were performed using a 2480 Wizard^2®^ automatic gamma counter from PerkinElmer (Waltham, MA, USA). Instant thin-layer chromatography (ITLC) strips (Agilent Technologies, Santa Clara, CA, USA) were used to estimate the radiochemical yield using a Cyclone^®^ Plus Storage Phosphor System from PerkinElmer (Waltham, MA, USA). The reversed-phase high-performance liquid chromatography (RP-HPLC) system we used was from Hitachi High-Tech (Tokyo, Japan), using a Luna C18 column (5 μm, 100 A°, 150 × 4.6 mm, Phenomenex, Værløse, Denmark) with a gradient from 5 to 70% acetonitrile (0.1% *v/v* trifluoroacetic acid) in water over 15 min. Sep-Pak^®^ C8 1 cc Vac cartridges (Waters Corp, Milford, MA, USA) were used for purification of the radiolabeled GRPR antagonist when needed.

The animal studies on biodistribution were approved by the Ethics Committee for Animal Research in Uppsala, Sweden following the national legislation on protection of laboratory animals (protocol code 5.8.18-00473/2021 approved on 26 February 2021). Acute toxicity experiments were approved by the Board of Medical Ethics, Tomsk National Research Medical Center of the Russian Academy of Sciences (protocol № 12 04.12.2020). 

### 2.1. Cell Culture

PC-3 cells were cultured in the RPMI-1640 medium supplemented with 20% fetal-bovine serum, 1% penicillin-streptomycin, and 1% L-glutamine. The cells were incubated at 37 °C and 5% carbon dioxide.

### 2.2. Kit Preparation and Radiolabeling

The kit for the clinical study was formulated as follows: freshly dissolved gluconic acid sodium salt (5 mg, in H_2_O), EDTA (100 µg, in H_2_O) and stannous chloride (75 µg, in 0.01 M HCl) were added to glass vials prior to direct addition of maSSS-PEG_2_-RM26 (40 µg, in H_2_O). The kits were prepared on ice and frozen at −20 °C directly after preparation. The kits were freeze-dried. Finally, the vials were sealed and sterilized by heat sterilization.

To radiolabel maSSS-PEG_2_-RM26, freshly eluted technetium-99m pertechnetate (^99m^TcO_4_^−^, 400–600 MBq) was added, in sterile saline, to the kit-containing vial followed by incubation at 90 °C for 60 min. The radiochemical yield was determined by ITLC and the ITLC strips were eluted with PBS (Rf = 0 for the radiolabeled peptide and Rf = 1 for ^99m^Tc-pertechnetate and ^99m^Tc-gluconate) and pyridine:acetic acid:water, 5:3:1.5 (Rf = 0 for hydrolyzed-reduced technetium colloid and Rf = 1 for the radiolabeled peptide, ^99m^Tc-pertechnetate and ^99m^Tc-gluconate). The reaction mixture was also analyzed using RP-HPLC. 

### 2.3. Cell Binding

The radiolabeled peptide binding to GRPR was tested in PC-3 cells (GRPR +) by the addition of 1 nM of [^99m^Tc]Tc-maSSS-PEG_2_-RM26 to the cells (~10^6^ cells, in triplicates), with and without pre-blocking of receptors, by the addition of 1 µM of NOTA-PEG_2_-RM26 (10 min at room temperature). After incubation at 37 °C for 1 h, the cells were treated with trypsin-EDTA, collected, and measured for their radioactivity content.

### 2.4. Validation of Kit Sterility 

The sterility of the lyophilized kit product was evaluated via growth promotion testing. Autoclaved growth medium, 30 g/L tryptic soy broth (TSB, Merck, Kenilworth, NJ, USA), was prepared in 200 mL flasks and incubated in 25 °C for 14 days either without or together with the kit sample. As reference samples, microorganisms starting at ~100 CFU were used. The reference microorganisms used were Candida albicans (C. albicans, ATCC^®^ 10231), Asperigillus brasiliensis (A. brasiliensis, ATCC^®^ 16404), and Bacillus subtilis (B. subtilis, ATCC^®^ 6633) (Thermo Fisher Diagnostics, Uppsala, Sweden). Culture growth was continuously monitored by visual inspection. The samples were considered sterile if no visible growth developed over the duration of the experiment. The suitability of the method was investigated by comparing parallel growth rates in between cultures with and without the added kit sample. For the method suitability tests, a kit sample buffer, lyophilized and reconstituted similarly to the kit product, was used. All experiments, except for the ones containing peptide sample, were performed in duplicates.

### 2.5. Toxicity Study

Acute toxicity experiments were carried out on 10 white outbred male rats (weight 220–260 g, age 10 weeks). The animals were kept in accordance with the European Convention for the Protection of Vertebrate Animals (Strasbourg, 1986) and the Rules of Laboratory Practice in the Russian Federation (Order of the Ministry of Health of the Russian Federation of August 23, 2010 N 708n), under standard conditions.

The maximum administered volumes of the medicine are justified by the standard recommendations for conducting preclinical studies, which stipulate that the maximum allowable volume of liquid for intravenous injection in rats is 2 mL. In an experimental group of rats (n = 5), the radiopharmaceutical with kit components was administered intravenously in the maximum volume possible for this method of administration: 2 mL, which amounted to 80 µg/kg. The control group (n = 5) received a similar volume of saline.

External examination after injection of radiopharmaceutical with kit components was carried out on the first day every 8 h, then daily for 14 d. During the experiment, the behavior, appearance, total weight, motor activity and reaction of animals to external stimuli were monitored.

### 2.6. Radiolabeling, In Vitro, and In Vivo Evaluation after Long-Term Storage

Randomly selected vials were stored at 4 °C at our laboratory for long-term testing. Within 18 months of storage, the vials were radiolabeled under the same conditions mentioned earlier. Additionally, the GRPR-binding in vitro was tested on a different batch of PC-3 cells, following the same protocol mentioned earlier. The in vivo GRPR-targeting properties of [^99m^Tc]Tc-maSSS-PEG_2_-RM26 were tested on PC-3 tumor-bearing Balb/c nu/nu mice (the cells (6 × 10^6^ cells/mouse) were inoculated into the mice 4 weeks prior to the experiment). On the day of the experiment, when palpable tumors developed, a group of mice (n = 4) was injected with [^99m^Tc]Tc-maSSS-PEG_2_-RM26 (30 kBq/40 pmol) and another group (n = 4) was additionally co-injected with NOTA-PEG2-RM26 (5 nmol) to block GRPR. The mice were euthanized 1 h post-injection and the organs of interest were collected, weighed, and measured for their radioactivity content.

Kit vials used in the clinical trial were stored at 4 °C at the hospital and labeled under the same conditions mentioned earlier for clinical use.

## 3. Results

**Figure 1:**
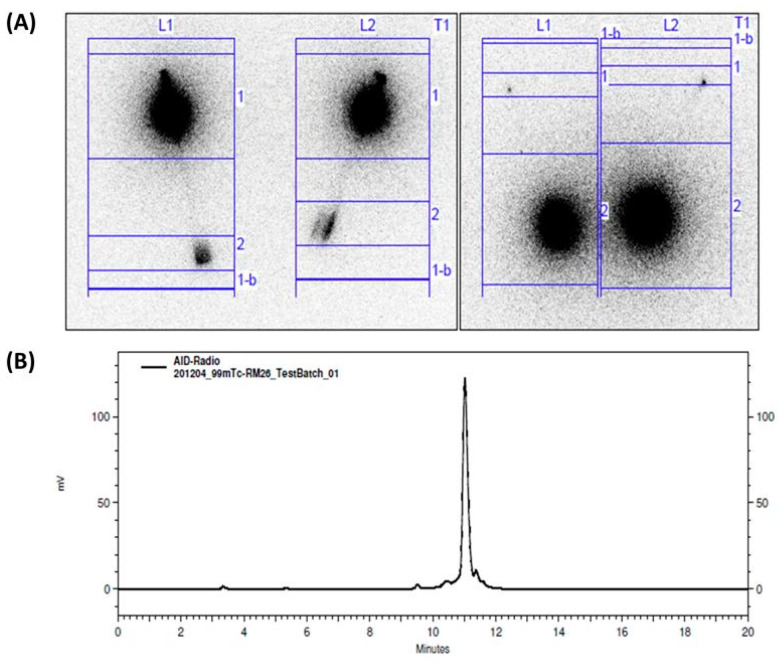
Analysis of labeling yield for [^99m^Tc]Tc-maSSS-PEG_2_-RM26 analyzed using (**A**) radioITLC (left: labelling yield, right: hydrolyzed-reduced technetium colloids) and (**B**) using HPLC. Activity distribution on the ITLC was measured using a PhosphorImager.

For freshly prepared kits, the radiochemical yield for [^99m^Tc]Tc-maSSS-PEG_2_-RM26 was >97%, while the level of hydrolyzed-reduced technetium colloids was below 0.5% (Figure 1).

**Figure 2:**
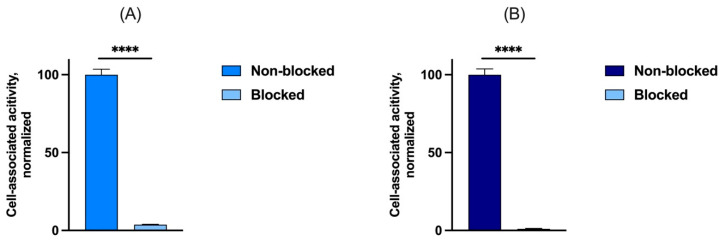
In vitro binding assay to validate the [^99m^Tc]Tc-maSSS-PEG_2_-RM26 prepared using (**A**) freshly prepared kit and (**B**) kit stored for 18 months. One nM of [^99m^Tc]Tc-maSSS-PEG_2_-RM26 was added with or without pre-blocking of GRPR in PC-3 cells. **** denotes a *p* value less than 0.0001.

The binding of [^99m^Tc]Tc-maSSS-PEG_2_-RM26 to GRPR was demonstrated by the significant difference of cell-associated radioactivity between non-blocked and pre-blocked receptors (*p* value < 0.0001, n = 3), as shown in Figure 2A.

Growth promotion tests revealed that the kit sample was sterile, since no growth could be seen in the growth media flasks 14 days after the addition of the sample. 

No animals died during the observation period when the kit’s constituents were tested for toxicity. Observation of the animals did not reveal any changes in their appearance, motor activity, or behavior. When weighing the rats at the end of the experiment (day 14 after administration), a uniform increase in body weight was revealed in all animals, while no toxic effect of the studied drug was detected.

**Figure 3:**
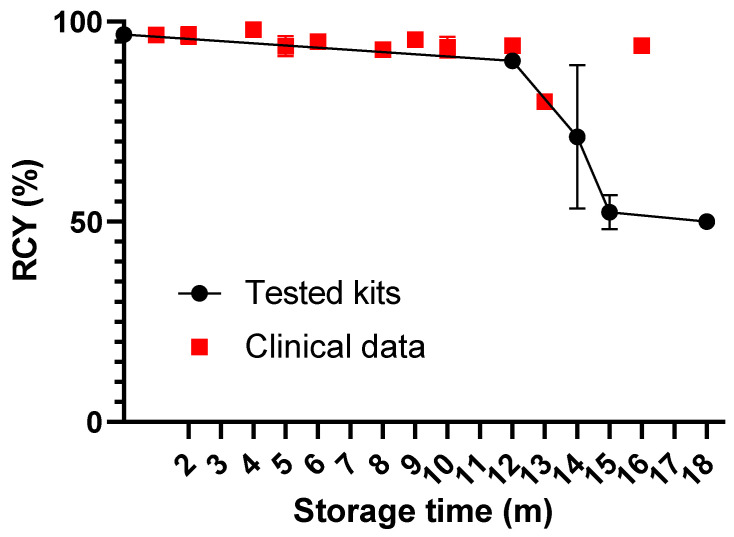
The change in radiochemical yield under 4 °C storage of the kit formulation: kit tested in laboratory (black circles) and kits used in clinical study (red squares). The error bars represent the standard deviation.

The labeling yields remained high when the kit vials were stored at 4 °C for one year. However, the further long-term storage (up to 18 months) resulted in stepwise decreased radiochemical yields (54 ± 3% for vials kept for 18 months), shown in Figure 3. The hydrolyzed-reduced technetium colloids remained low (<0.5%) over time. Upon purification using Sep-Pak cartridges, the radiochemical purity of [^99m^Tc]Tc-maSSS-PEG_2_-RM26 was >97%. The binding of the purified [^99m^Tc]Tc-maSSS-PEG_2_-RM26 to non-blocked GRPR on PC-3 was significantly higher than the uptake on PC-3 cells with pre-blocked GRPR, as seen in Figure 2B.

The labeling yields obtained in the clinical setting corroborated the yields for control vials (Figure 3).

**Figure 4:**
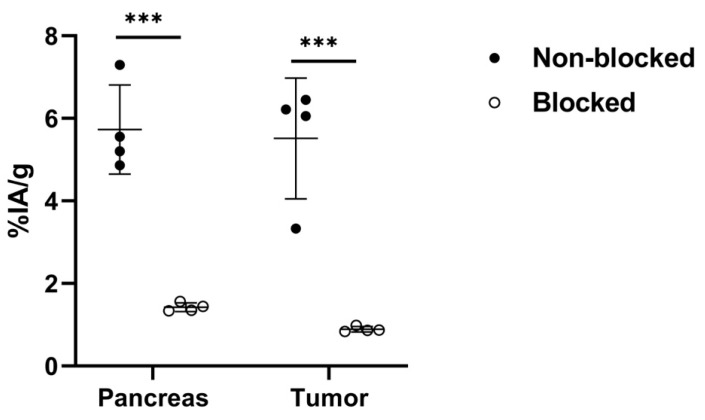
In vivo targeting of GRPR in Balb/c nu/nu mice bearing PC-3 tumors after 1 h of intravenous injection of [^99m^Tc]Tc-maSSS-PEG_2_-RM26. *** denote a *p* value less than 0.001.

The biodistribution pattern of [^99m^Tc]Tc-maSSS-PEG_2_-RM26 (Table A1) was obtained using a kit stored for 18 months and was similar to that reported for [^99m^Tc]Tc-maSSS-PEG_2_-RM26, which was labeled through a multiple-step procedure [20]. The in vivo GRPR-targeting study, shown in Figure 4, demonstrated a 6-fold difference in PC-3 tumor uptake (*p* < 0.001, n = 4). There was also a 4-fold difference in pancreatic uptake between non-blocked and blocked groups (*p* < 0.001, n = 4).

## 4. Discussion

GRPR targeting for the imaging of prostate cancer has been increasingly evaluated in recent years due to the limitations of the well-established PSMA targeting, which include PSMA-negative lesions [24], false-positive scans [25], and a degree of resistance to PSMA-targeted radionuclide therapy [26]. To provide wider coverage, a need for imaging prostate cancer by targeting cell markers other than PSMA is present, and GRPR has emerged as an important target especially for oligometastatic prostate cancer.

**Figure 5:**
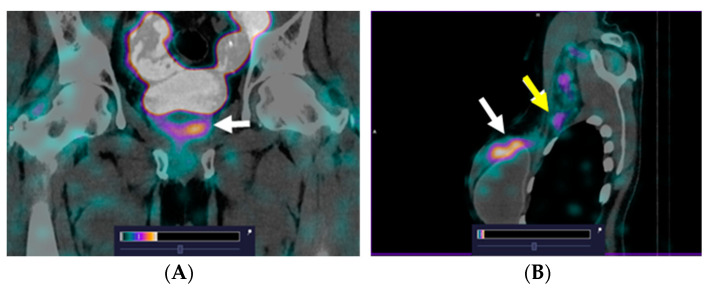
Fused SPECT/CT images of prostate cancer (**A**) and breast cancer (**B**) patients 2 h after injection of [^99m^Tc]Tc-maSSS-PEG_2_-RM26. A focus of increased [^99m^Tc]Tc-maSSS-PEG_2_-RM26 uptake is visualized in the prostate gland ((**A**), white arrow), in the right breast ((**B**), white arrow), and in the right axillary node ((**B**), yellow arrow).

We previously reported the preclinical evaluation of maSSS-PEG_2_-RM26, radiolabeled with technetium-99m with high radiochemical yields, which demonstrated strong affinity for GRPR (equilibrium dissociation constant in the low nanomolar range). Furthermore, it also showed a biodistribution profile suitable for GRPR-positive prostate cancer imaging and acceptable dosimetry estimations when tested on animal models [20]. Recently, [^99m^Tc]Tc-maSSS-_PEG2_-RM26 was studied in a phase I clinical study, and it was demonstrated that single injections of this agent were well tolerated and were associated with low absorbed doses in healthy organs and tissues [27]. The agent was tested for SPECT imaging of prostate and breast cancer lesions, and a number of primary tumors of both origins, as well as breast cancer lymph node metastases, which were visualized shortly after administration (Figure 5).

The preparation of technetium-99m-labeled radiopharmaceuticals for clinical use is often a multistep process that can be time-consuming and can also be associated with an increased risk of human error. Therefore, the use of a kit formulation that enables one-pot single-step labeling, using freshly eluted ^99m^TcO_4_^−^, will shorten the overall time needed for preparation and reduce the risk of human error by simplifying the preparation of the radiopharmaceutical. Following the kit formulation reported by Ahlgren et al. [22], our kits were formulated to contain maSSS-PEG_2_-RM26, gluconic acid sodium salt, EDTA, and stannous chloride. The constituents were lyophilized shortly after preparation to avoid the risk of hydrolysis of stannous chloride, which will in turn result in a dramatic decrease, if not a total loss, of radiochemical yields after radiolabeling with ^99m^TcO_4_^−^.

After lyophilization and sterilization of the kit constituents, further testing of the labeling efficacy and binding specificity to GRPR was necessary to ensure preservation of the characteristics of [^99m^Tc]Tc-maSSS-PEG_2_-RM26. The kit constituents were not affected by the preparation and heat sterilization. This was reflected by the consistently high radiochemical yields which were indifferent to multistep labeling. The significantly higher cell-associated activity for unblocked GRPR than blocked GRPR on PC-3 cells indicated that [^99m^Tc]Tc-maSSS-PEG_2_-RM26 maintained its high specificity for GRPR. Sterility testing of the kit constituents indicated that the kit was free from possible contaminants; this is an important step in deeming a pharmaceutical product safe for use in clinics. Additionally, the toxicity studies did not result in any negative effect on the animals, indicating that the kit constituents are not toxic to animals and that toxicity to humans is not to be expected.

The use of the one-pot single-step radiolabeling of maSSS-PEG_2_-RM26 with generator-eluted ^99m^TcO_4_^−^, provides the possibility to use [^99m^Tc]Tc-maSSS-PEG_2_-RM26 in a clinical setting directly after radiolabeling and radiochemical yield analysis without further processing. This adds great value to a clinical setting by simplifying preparation of the imaging probe overall and thereby leading to a more robust and safe production. Particularly, this is accomplished by minimizing the steps required from eluting technetium-99m to injecting [^99m^Tc]Tc-maSSS-PEG_2_-RM26 for SPECT imaging, reducing the overall time of preparation, reducing the handling time of the activity, and reducing the risk of human error and other errors related to the radiopharmaceutical preparation. Together, these actions should also lead to a more cost-efficient production of a radiolabeled probe. Many cancer patients could benefit if a SPECT tracer with simple and robust preparation were implemented in a clinical practice. Prostate cancer patients with localized or oligometastatic disease, in which lesions could have low PSMA expression, could be tested as negative by using a PSMA scan. However, the majority of these patients could have GRPR expression (>75% in primary prostate cancer and >85% in lymph node metastases), as GRPR expression is associated with small lesions in low-grade prostate cancer [9,10,28]. In addition, this tracer can be used for molecular characterization of patients with hormone-driven breast cancer, where >80% of estrogen receptor positive lesions also express GRPR [29,30].

Long-term storage of pharmaceuticals can affect their stability and quality. The storage of our kit formulation at 4 °C for 18 months impacted the radiochemical yields of [^99m^Tc]Tc-maSSS-PEG_2_-RM26, therefore, an additional purification step was necessary when kits older than 14 months were used. However, the high radiochemical purity of [^99m^Tc]Tc-maSSS-PEG_2_-RM26 was achieved upon purification. Furthermore, analysis of the GRPR-binding properties, both in vitro and in vivo, demonstrated retained, high specificity to GRPR. We could speculate that water from the storage environment, that could humidify the kit vials or remaining water in the kit vials, could hydrolyze stannous chloride and thereby lead to lower labeling yields with time. The addition of freshly prepared stannous chloride to aged kits restored labeling efficacy (data not shown). Furthermore, we plan to perform an evaluation of storage at −20 °C and investigate how this affects the labeling performance.

## 5. Conclusions

We have developed a lyophilized kit for one-pot single-step labeling of the GRPR antagonist, maSSS-PEG_2_-RM26. Imaging probe [^99m^Tc]Tc-maSSS-PEG_2_-RM26 produced using the lyophilized kit was stable and resulted in a biologically functional imaging agent for SPECT examination of GRPR expression. The kit formulation can be stored for up to one year at 4 °C without any effect on the radiochemical yields or the biological characteristics of the radiotracer. A purification step may be necessary if the radiochemical yields are not sufficient, and this procedure does not influence the biological properties of the product. 

## 6. Patents

Patent 2776234 C1 (14.07.2022, application № 2021124249 12.08.2021), Russian Federation.

## Data Availability

The data generated during the current study are available from the corresponding author upon reasonable request.

## Appendix A

**Table A1:** Ex vivo biodistribution of [^99m^Tc]Tc-maSSS-PEG_2_-RM26 in Balb/c nu/nu mice bearing PC-3 xenografts 1 h pi. Non-blocked group was injected with 40 pmol of peptide labeled with 30 kBq of technetium-99m, blocked group—with the same amount of labeled peptide and 5 nmol of NOTA-PEG2-RM26. Data are presented as average values of % of injected activity per gram (%IA/g) ± SD (n = 4).

Organ	Non-Blocked	Tumor-to-Non-Tumor Ratios for Non-Blocked Group	Blocked
Blood	0.3 ± 0.2	30 ± 4	0.3 ± 0.4
Liver	2.9 ± 0.8	2.0 ± 0.4	1.7 ± 0.4
Spleen	0.13 ± 0.05	51 ± 18	0.13 ± 0.06
Pancreas	6 ± 1	1.1 ± 0.2	1.4 ± 0.1
Kidneys	5.0 ± 0.4	1.3 ± 0.2	5.0 ± 0.6
Tumor	6 ± 1	–––	0.89 ± 0.07
Muscle	0.05 ± 0.02	125 ± 50	0.032 ± 0.008
Bone	0.09 ± 0.05	78 ± 42	0.042 ± 0.009
